# From Network Analysis to Functional Metabolic Modeling of the Human Gut Microbiota

**DOI:** 10.1128/mSystems.00209-17

**Published:** 2018-03-27

**Authors:** Eugen Bauer, Ines Thiele

**Affiliations:** a Luxembourg Centre for Systems Biomedicine, Universite du Luxembourg, Esch-sur-Alzette, Luxembourg; Argonne National Laboratory

**Keywords:** computational modeling, constraint-based modeling, gut microbiome, metabolic modeling, network approaches

## Abstract

An important hallmark of the human gut microbiota is its species diversity and complexity. Various diseases have been associated with a decreased diversity leading to reduced metabolic functionalities. Common approaches to investigate the human microbiota include high-throughput sequencing with subsequent correlative analyses. However, to understand the ecology of the human gut microbiota and consequently design novel treatments for diseases, it is important to represent the different interactions between microbes with their associated metabolites. Computational systems biology approaches can give further mechanistic insights by constructing data- or knowledge-driven networks that represent microbe interactions. In this minireview, we will discuss current approaches in systems biology to analyze the human gut microbiota, with a particular focus on constraint-based modeling. We will discuss various community modeling techniques with their advantages and differences, as well as their application to predict the metabolic mechanisms of intestinal microbial communities. Finally, we will discuss future perspectives and current challenges of simulating realistic and comprehensive models of the human gut microbiota.

## Abstract (precis)

An important hallmark of the human gut microbiota is its species diversity and complexity. Various diseases have been associated with a decreased diversity leading to reduced metabolic functionalities.

## INTRODUCTION

Microbial communities are abundantly present throughout nature and form many beneficial symbiotic interactions with different eukaryotic hosts. In many cases, the host acquires novel functionalities with mutualists that can contribute to host fitness and well-being (1) by, e.g., the supplementation of essential nutrients (2). These functional microbes can be found on the interface of nutrient absorption in the intestinal ecosystem (3) and gut-associated structures, such as bacteriomes (4). Removal of these symbiotic microbes can lead to host fitness decrease, demonstrating the host dependence on the symbionts (2) and the relevance of symbiotic interactions.

While the symbiosis between the human gut ecosystem and its host is less well understood than other symbioses, intestinal microbial communities have been associated with human well-being (5). In particular, microbial metabolism is considered relevant for nutrient provisioning and complementary digestion of food (6). The human gut microbiota consists of more than thousands of microbial species (7), which can form manifold metabolic interactions between themselves and with their host. Interestingly, the microbiota metabolism is more conserved between human individuals than the species composition, which suggests that redundant functionalities are present in these microbes, complementing each other (8). The metabolic functions of various gut microbes are suggested to benefit human health with the provisioning of vitamins (9) and fermentation products (10). Microbial fermentation products can be utilized by the human host as an additional energy source (11) and benefit the immune system (12), therefore playing a pivotal role for human well-being.

A loss of microbial and metabolic diversity can lead to various microbiota-associated diseases, such as obesity (13), type 2 diabetes (14), and inflammatory bowel disease (IBD) (15). Treatments for gut-associated diseases also aim to modulate the human gut microbiota such that it exhibits healthy characteristics. Such treatments can include fecal microbiota transplantation (16), probiotics (17), and dietary change in the form of prebiotics (18). Since the microbiota varies between individuals, treatments are ideally personalized to support each patient’s unique needs. Consequently, it is important to understand the mechanism by which the microbiota is influenced to thereby enable the design of novel treatment with higher efficacies.

With the advent of high-throughput sequencing technologies, we can now enumerate and describe the microbial and functional diversity of the human gut with respect to different diseases and conditions. These analyses have unraveled a complex microbial ecosystem that is influenced by diet and environmental factors (19). Furthermore, transcriptomic and proteomic analyses have allowed researchers to probe the metabolic activity of the microbiota, revealing high activity of fermentative pathways and carbohydrate utilization (20). Whole-genome sequencing of single microbes can give hints on the functions and capabilities of individual members of the intestinal microbial community (21). Recently, it has become possible to reconstruct individual genomes from metagenomic data (22). Thus, omics analyses have broadened our understanding of the human gut microbiota in terms of the possible metabolic function and microbes that occur in this complex ecosystem. However, it is still difficult to assess the ecology in terms of metabolic interactions between microbes and how each microbe contributes to the intestinal microbial community.

To understand the ecological mechanisms that drive the human gut microbiota better, it is important to go beyond the descriptive nature of high-throughput data analysis. This can be achieved by formulating knowledge- or data-driven models, which can describe the underlying ecosystem and give mechanistic insights for hypothesis generation and designing subsequent experiments. A recent review described the relevance of such models in giving novel causal relationships in a field that is dominated by data and correlation (23). Here, we will discuss current systems biology approaches that have been applied to the human gut microbiota. As highlighted above, metabolism is one of the key features in the gut microbiota and microbes in general; we will thus focus on this aspect. First, we outline network topology analyses that are based on newly generated data or already existing knowledge. Then, we will describe modeling approaches in the field of constraint-based modeling that allow researchers to simulate interactions within microbial communities. Finally, we will highlight the advantages and limitations of these approaches and give suggestions for further studies.

## NETWORK-BASED APPROACHES APPLIED TO THE HUMAN GUT MICROBIOTA

**FIG 1:**
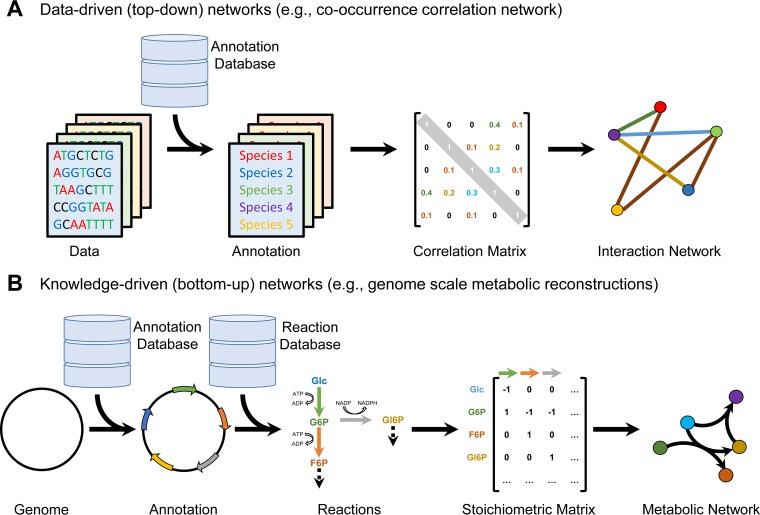
Examples of data-driven network reconstruction based on high-throughput data from different patients or conditions (A) and knowledge-driven networks based on genome-scale metabolic reconstructions (B).

Network-based approaches are used to identify relevant microbes or metabolites of the human gut microbiota. Networks are usually represented by interactions in the form of edges that connect biological components in the form of nodes. With respect to the human gut microbiota, these components can represent species that interact (24) or metabolites that are converted through biochemical reactions (25). The goal of such networks is to provide a global overview of the underlying system and possible mechanisms, which makes it possible to identify relevant microbes or metabolites that play important roles in the network by connecting a variety of components or exhibiting key features. In the next paragraphs, we will discuss networks that are created top-down with newly generated data and knowledge-derived networks that are created bottom-up with existing information (Fig. 1).

### Data-driven (top-down) networks.

Species cooccurrence networks can be used to investigate ecological interactions between microbes. Based on high-throughput data analyses, simple networks can be constructed with the species abundance information for different patients (24). Such networks contain the information of cooccurring species based on calculation of correlation coefficients for each pair of microbes (Fig. 1A). Consequently, a positive interaction can be deduced if two microbes have a positive correlation, e.g., cooccur with each other, and a negative interaction can be deduced if the coefficient is negative, e.g., excludes the presence of each other (24). This information can be relevant for understanding ecological concepts that drive exclusion mechanisms and niche differentiation. Furthermore, the information on species that interact positively or negatively can enable further experimental studies to investigate mechanisms by which these interactions may take place, e.g., production of antibiotics or metabolic cross-feeding. Despite their benefits and merits, such network-based approaches have the limitation that they do not provide hypotheses on potential mechanisms for the species interactions.

To overcome these limitations, metabolism-based networks have been used to assess the functional aspect of the human gut microbiota. Therefore, metabolic functions or enzymes annotated from high-throughput sequencing of different human individuals can be used to identify pathway or metabolite differences (26). By connecting enzymes based on the reactions that they are involved in, it is possible to construct metabolic networks specific for each patient (26). Topological analyses of these networks can reveal patient-specific differences and global differences between healthy controls and patients (26). Additional information can be gained by finding differences in enzymatic modules consisting of multiple related metabolic functions, which are connected and thus influence each other. This information can be important when trying to find potential components for treatments or assessing off target effects (26). While such networks are primarily constructed from patient data, the connections between the reactions are retrieved from previous knowledge, e.g., biochemical reaction databases (Fig. 1).

### Knowledge-driven (bottom-up) networks.

In the bottom-up network approach, biochemical knowledge is used to generate specific networks, e.g., biochemical networks, from information provide in databases, such as the Kyoto Encyclopedia of Genes and Genomes (KEGG) (27), MetaCyc (28), and ModelSEED/Kbase (29, 30). In a subsequent step, the high-throughput data can then be overlaid with such networks to provide novel insight and facilitate the generation of hypotheses. For instance, biochemical reaction information has been used to construct a global view of the metabolic reactions and pathways that take place in the human microbiota to assess its overall metabolic capacity (20). Useful visualizations of these networks can be achieved by maps representing how reactions interact with each other and share metabolites (20). By subselecting these maps according to patient data, it is possible to find differences in terms of various gut locations (20). With these maps, it is possible to link metabolic functions and generate a global overview; however, the information on which metabolic functions are carried out by specific microbes and how they interact is missing in such analyses.

In another study, the metabolic exchange between microbes has been represented in a global interaction map, which helped the identification of metabolic deficiencies in certain diseases (31). On the basis of data in databases and scientific literature, microbes have been represented by their known transport of various metabolites, which can act in a beneficial or detrimental manner to the cooccurring species. The global view of all transports gave clues as to which nutrients were converted by specific microbes and then potentially supplemented to the host. When mapping patient data to this network of transporters, it was possible to identify potential exchange deficiencies compared to healthy controls (31). While these analyses can reveal potential metabolites or interactions that are differentially regulated in patients, they lack the view on the complete metabolism of each microbe species.

In a complementary approach, genome-scale metabolic reconstructions of organisms are constructed based on genomic and biochemical data to represent comprehensively their metabolism to investigate the metabolic potential. To facilitate the reconstruction process, available automatic reconstruction pipelines, such as ModelSEED (29), can be used to generate an initial reconstruction based on the genome annotation, which can be, e.g., retrieved from RAST (32). On the basis of the annotation of each gene, enzymes are predicted, which carry out one or multiple reactions. In the automated reconstruction process, biochemical reactions are then retrieved from a database, such as KEGG (27) or MetaCyc (28). Subsequently, biochemical reactions are represented in a stoichiometrically accurate manner by their metabolite educts and products and their thermodynamic directionality (reversible or irreversible). To ensure correct reaction stoichiometry (33) and directionality (34, 35), further curation of the database entries may be necessary (36) (see also below). The comprehensive set of reactions retrieved by this process constitutes the metabolic reconstruction and are mathematically represented in form of the stoichiometric matrix (S-matrix) (Fig. 1). The rows in the S-matrix represent the metabolites, and the columns represent the biochemical reactions. Entries are stoichiometric coefficients for each metabolite participating in a reaction. Through sharing different metabolites, reactions are connected with each other and therefore represent a metabolic network. In a recent study, we applied the automatic pipeline of ModelSEED (29) to reconstruct 300 representative microbes that are present in the human gut (37). We compared the metabolic networks with each other to find taxon-specific differences between the gut microbes. On the basis of this analysis, we found that microbial strains can be more metabolically different than predicted by phylogeny, which highlights the need for taking a diversity of microbes into consideration to understand the complete metabolism of the human gut microbiota.

While the automatic reconstruction process provides a good approximation of a species’ metabolism, the manual curation of reactions is essential for metabolic modeling. On the basis of biochemical knowledge of reaction directionality and substrate uptake, reactions are refined within the metabolic reconstruction to be more congruent with experiments (36). This manual effort cleans up mistakes that are still prevalent in automatic reconstructions and further expands the metabolic network for biologically relevant simulation. Several resources and databases are available with various degrees of curation status (Table 1). In a recent publication (25), we retrieved automatically reconstructed gut microbe models and applied manual curation efforts to provide a resource for further refinements and metabolic modeling efforts.

**TABLE 1:** Resources and databases to retrieve genome-scale metabolic models of human gut microbes with their respective curation status

Resource	Curation status	No. of microbe reconstructions	Reference
Kbase^a^	Draft		30
MetaCyc^a^	Draft		28
ModelSEED^a^	Draft		29
AGORA	Curated	773	25
BiGG database	Curated	78	38
Human metabolic atlas	Curated	5	39

^a^ Any available genome sequence can be uploaded to reconstruct a draft metabolic network.

## CONSTRAINT-BASED MODELING OF INTESTINAL MICROBIAL COMMUNITIES

### Constraint-based reconstruction and analysis. 

**FIG 2:**
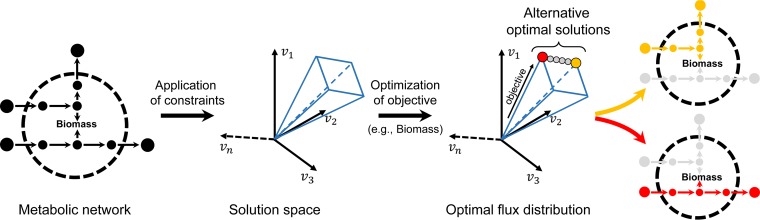
Flux balance simulations of individual genome-scale metabolic models and the emergence of alternative optimal flux distributions.

Constraint-based reconstruction and analysis (COBRA) is based on genome-scale metabolic reconstructions, which are formalized as metabolic models to simulate biological relevant physiological states. By applying condition-specific constraints, reconstructions are converted into metabolic models (Fig. 2). Often, a particular linear metabolic objective is defined, e.g., a biomass reaction, which summarizes all biochemical precursors known to be required to form a new cell or organism. The following mathematical problem can then be formulated while assuming the biological system to be at a steady state: maximize *v*_*B*_, which is subject to *S* × *v* = 0, and *v*_*i*,min_ ≤ *v*_*i*_ ≤ *v*_*i*,max_, where *v* represents the flux values (minimum and maximum values) through all model reactions *i* while maximizing the flux *v*_*B*_through the metabolic objective reaction B. The steady-state assumption implies that metabolites cannot accumulate and the total flux into the network must equal the total outflux, and it is represented by *S* × *v* = 0, where *S* is the aforementioned stoichiometric matrix. The constraints, represented as *v*_*i*,min_ ≤ *v*_*i*_ ≤ *v*_*i*,max_, can reflect medium conditions, in which the uptake of metabolites via exchange reactions is limited or limitations of internal reaction fluxes that come from experimental data (36). The biomass flux then predicts how much biomass the organism can produce under the given condition. Fluxes that flow through the network predict the metabolic pathways that are used by the organism in order to achieve the metabolic objective. The process of finding the solution to the stated optimization problem is called flux balance analysis (FBA) (40) and can be solved using linear programming. To help with the interpretation and data integration, fluxes are typically scaled to millimoles per gram (dry weight) per hour, and the biomass is usually given in gram (dry weight) per hour (41). Constraint-based modeling approaches can be applied to the human gut microbiota by either analyzing the metabolic capabilities of single species (42–45) or combining metabolic models of multiple species in a community modeling approach (46–50). Computational toolboxes, such as the COBRA toolbox (51), facilitate such investigations by the systems biology and the microbiome research communities.

An advantage of the COBRA approach is that it can assess the spectrum of metabolism for an organism (52). The steady-state solution space contains all possible flux distributions consistent with the applied constraints for a given condition-specific model. To deal with this degenerative nature of FBA, i.e., that there are typically more than one possible flux distribution, parsimonious FBA can be used to select the flux distribution that minimizes the sum of all reactions fluxes and thereby estimates the minimization of enzyme usage (53). This particular flux distribution can be computed with an additional linear programming problem, which minimizes the total flux while ensuring the calculated biomass objective (53). Furthermore, flux variability analysis (FVA) (54, 55) can be applied, in which each reaction in the model is minimized and maximized with the aim to find the range of flux values for each reaction (i.e., the flux span) that can be carried by each reaction. These computations enable the overall assessment of metabolic functionalities an organism can achieve.

### Compartmentalized community models.

**FIG 3:**
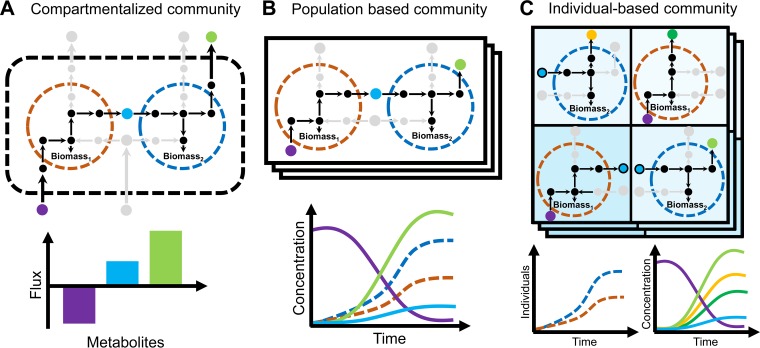
Flux balance simulation (FBA) of compartment-based communities (A) as well as dynamic FBA of population-based (B) and individual-based models (C).

Compartmentalized microbial community models can be constructed by integrating individual microbial metabolic reconstructions via their S-matrices (Fig. 3A). In this combined model, the individual microbes are separated from each other by occupying different compartments, in which they can secrete and take up metabolites from a shared environment (56). Therefore, the microbes can compete for nutrients in this environment but can also support each other by releasing metabolites to be used by other microbes. The optimized biomass of this community is usually composed as a combination of the individual biomass reactions of each microbe (56). Additionally, coupling constraints can be applied to ensure that the flux through each biochemical reaction is scaled with the achievable biomass reaction (57). Further developments of the community objective include a multiobjective optimization, in which the egoistic growth interest of each microbe is optimized while ensuring an overall community growth (58). These methods allow the prediction of metabolic interactions based on combined S-matrices.

Pairwise models of microbial communities can give new insights in terms of the metabolic interactions that occur in the human gut. In a recent study, we applied pairwise community models to the human gut microbiota by modeling all possible pairs of more than 700 microbes and found strikingly mostly negative metabolic interactions, e.g., competition for resources, of different microbes (25), which underlines the ecological concept that negative interactions shape diversity in the human gut microbiota (59). Several other studies have investigated pairwise interactions of gut bacteria and discovered novel metabolic interactions and the exchange of key metabolites (57, 60, 61). By simply adding additional microbes to the compartment-based strategy, it is possible to model intestinal microbe communities of multiple species (49). A study based on 11 metabolic models (62) revealed a vivid exchange of fermentation products and the conversion of metabolites involved in neurotransmitter production, which can have important implications in various neurological diseases. The concept of modeling multiple species has been further applied to model the influence of different diets on the metabolic interactions of different gut bacteria (63). While such generated information of compartmentalized community models can be useful in finding new hypothesis of metabolic interactions, they do not represent the temporal dynamics of the interactions between the species.

### Population-based models.

Population-based COBRA modeling can be used to analyze temporal dynamics of metabolic interactions within microbial communities. With the optimization of biomass growth, FBA intrinsically models growth of microbe populations. The combined S-matrix approach can also be used to model population dynamics with respect to a temporal scale (64). Essentially, the biomass optimization of each microbe is solved iteratively and independently. Each iteration represents a discrete time interval, in which new biomass (as defined by the biomass reaction) is produced and metabolites are secreted or taken up. The units of the fluxes and biomass are scaled according to the time interval. Similar to the compartmentalized community models, metabolites are secreted into a shared compartment. Therefore, the same positive or negative interactions between microbes, as discussed above, can potentially take place. However, through the introduction of a temporal scale via the time intervals, it is possible to investigate the evolution and emergence of these interactions under different conditions. This extension can help with the understanding of how different interactions are temporally dependent, as it is thought to be the case for the human gut microbiota where nutrient intake is dynamically changing throughout the day (65). Experimental time series data can be integrated in such temporal dynamics to adjust the simulation results (66). Recent approaches also add a spatial dimension to such community models to represent the colony growth of organisms (67). The inclusion of a spatial dimension allows the representation of metabolite concentrations, which can diffuse and create different gradients. Such gradients are thought to strongly influence the gut microbiota by forming different niches in which the microbial community differentiates (68). Population-based community modeling can therefore help to investigate what metabolites affect and dynamically change the microbial community.

Population-based COBRA modeling can give new insights in the dynamic change of metabolic interactions of human gut microbial communities. A recent approach applied population-based metabolic models to a simple community of six microbes (69). The results of this analysis demonstrated a highly metabolic active community that exchanges a variety of different metabolites through a complex interaction network. Within this interaction network, many cross-feeding interactions have been validated against knowledge from literature. Notably, these interactions can change over time and are therefore highly dynamic (69). By calibrating the relative abundances of a representative intestinal microbial community, a recent study (70) could also reproduce the dynamic change of the community structure in response to different diets. A significant assumption of such models is that each microbe consists of a population of homogenous cells, which operate under similar environmental niche conditions. This approach can be useful to investigate the metabolic behavior of microbial populations but is limited in predicting metabolic interactions between individuals of one population.

### Individual-based community models.

The metabolic interactions between distinct microbial organisms can be modeled by combining the COBRA approach with individual-based modeling (IBM). IBM is a method also used in classical ecology (71) to model species populations in terms of single independent individuals by discrete time steps and a spatial environment. Individuals in this environment can interact according to predefined rules, which determine their states. Through these local interactions, global population structures can emerge that determine the system state. Therefore, population dynamics can help to understand how single cells shape and structure populations of species in an ecosystem. Several approaches have been published that combined IBM with COBRA modeling to study the metabolism of single cells in a population of a single species (72) or multiple species (73, 74). Briefly, each single cell is represented by a metabolic model and simulates its metabolism according to the spatial position in the environment, which includes certain metabolites. Metabolites can diffuse through this environment, described using partial differential equations, to reflect concentration gradients that dominate the community and to create niches that allow the activation of different metabolic pathways. Each species is thus represented by metabolically heterogeneous individuals, which permits the simulation of the full metabolic potential (73). Thus, this method allows researchers to predict the metabolic interactions between species and within species that can have different metabolic phenotypes depending on the spatial resource allocation (Fig. 3C).

Individual-based COBRA modeling can give insights into the spatial and temporal community structure of the human gut microbiota. For instance, a recent study has explored the effects of antibiotics on a two-species community model of human gut microbes, represented by a combination of individual-based and kinetic modeling (75). Similarly, the combined approach of IBM and COBRA has been applied to analyze microbial communities in the human gut (73). The community model contained seven representative microbes that have previously been experimentally shown to represent the properties of the gut microbiota (76). The simulations recapitulated experimentally known metabolite concentrations but also predicted novel cross-feeding interactions through fermentation products exchanged within the microbes of the community (73). Furthermore, by applying a spatial gradient of mucous glycans, spatial niche differentiation of microbial cells could be observed, consistent with experimental microscopy studies. This observation further strengthens the fact that metabolism is an important factor in shaping the gut microbiota and inducing ecological interactions. Recently, this approach has been further expanded to integrate metagenomic data of patients and healthy controls to predict personalized dietary treatments (77). Such findings demonstrate the relevance of the integration of ecological methods with COBRA to understand the metabolic mechanisms that shape the temporal and spatial community structure. There are several tools available that allow constraint-based modeling of microbial communities (Table 2).

**TABLE 2:** List of available tools as freely accessible software packages for constraint-based modeling of microbial communities

Strategy and tool	Tutorial(s)	Link	Reference
Compartment-based models			
MMinte	Yes	https://github.com/mendessoares/MMinte	61
OptCom	No	http://www.maranasgroup.com/submission/OptCom.htm	58
Microbiome modeling toolbox	Yes	https://opencobra.github.io/cobratoolbox/	51
Population-based models			
COMETS	Yes	http://www.bu.edu/segrelab/comets/	67
MCM	Yes	http://www.zoology.ubc.ca/MCM	66
DyMMM	No	https://sourceforge.net/p/dymmm	64
SteadyCom	No	https://github.com/maranasgroup/SteadyCom	70
Individual-based model			
BacArena	Yes	https://github.com/euba/BacArena	73

## CURRENT CHALLENGES OF GUT MICROBIOTA MODELING

The COBRA approaches for analyzing microbial communities provide promising tools for investigating the metabolic effect of the human gut microbiota. Several studies have been conducted in this context, mostly focusing on small communities representative of the intestinal microbiota (Table 3). These studies have revealed several important aspects of the human gut microbiota and its metabolism through the use of mechanistic metabolic models, which allow researchers to identify relevant metabolic pathways underlying the observed metabolic interactions between species. These analyses can improve our understanding of the metabolic mechanisms that shape intestinal microbial communities, but there are several limitations and challenges that need to be considered when applying COBRA community modeling to the human gut microbiota.

**TABLE 3:** List of the different constraint-based community modeling approaches that have been applied to model microbial consortia of the human gut microbiota

Strategy and application to the human gut microbiota	No. of microbial species	With host	Reference
Compartment-based models			
Host-microbe metabolic interactions	1	Yes	57
Microbe-microbe metabolic trade-off	2	No	60
Microbe-microbe metabolic interactions	3	No	48
Microbe-microbe metabolic interactions with different diets	4	No	63
Human metabolic interactions with microbial community	11	Yes	90
Metabolic interactions between microbes in community	11	Yes	62
Metabolic interactions between microbes in community, emergent metabolic properties of personalized microbial communities	>100	No	50
Metabolic interactions between personalized microbial communities and whole-body and organ-level metabolism	>100	Yes	81

Population-based models			
Dynamic metabolic interactions within microbial community	6	No	69
Simulating microbe abundance profiles	9	No	70

Individual-based models			
Effects of antibiotic treatments on the metabolic interactions between species	2	No	74
Diet interactions within microbiota and cross-feeding	3	No	74
Niche differentiation induced by mucous glycans	7	No	73
Integration of metagenomic data and prediction of personalized dietary treatments	>100	No	77

### Horizontal gene transfer.

A challenging aspect in community modeling of the human gut microbiota is horizontal gene transfer (HGT). HGT seems to occur frequently within the human gut microbiota (78, 79) and represents a source for evolutionary innovation. Though the effect of HGT on gut-associated community has not been studied yet, metabolic models could be used to address such questions (80). Thus, the combination of evolutionary and ecological modeling could give novel insights into the dynamics of the human gut microbiota.

### Scalability and model complexity.

One of the most striking hallmarks of the human gut microbiota is its species diversity, which poses challenges to the model simulations that need to be addressed. Simplified microbiota models of less than 10 species are relevant for studying metabolic interactions in general and can be used to simulate experiments that are conducted with small microbial communities in gnotobiotic animals (76) or *in vitro* (82). However, such models will never be able to capture and explain the high complexity of the human gut microbiota. Why are there so many different species? Why is the human gut microbiota more diverse than other body sites? Why are some diseases associated with a lower microbiota diversity? These are some of the questions that can be addressed only with more comprehensive microbiota model. In a recent publication (25), we created a resource of more than 700 curated metabolic models of gut microbes. Combining those into a community model poses several difficulties, such as the simulation time. Compartmentalized models are extended versions of single metabolic models, which become more challenging to solve as the number of variables and mass-balances increases. In particular, extensive calculations associated with simulation methods, such as FVA, require dedicated algorithms that make effective use of, for instance, parallelization of the computations (55, 83). By doing so, we were able to simulate with microbiota and host-microbiota models containing hundreds of thousand variables and linear equations (50, 81), demonstrating the scalability of this approach. Population-based models, on the other hand, simulate each microbe independently and scale therefore with the number of species in the community. Individual-based community models are generally independent of the number of species but scale linearly with the number of individuals (73) and are therefore limited in modeling a small spatial scale. Taken together, each simulation approach requires the consideration of the trade-off between model complexity and simulation time.

Model complexity also poses the problem of subsequent data analysis and visualization. By simulating large-scale microbiota models with a high number of variables, it becomes difficult to identify the explaining pathways. The large amount of simulations and the complexity of the models further require data analysis approaches to find the most relevant parameters influencing the modeled system with the aim to propose an experimentally testable hypothesis. State-of-the art machine learning approaches have already been used to aid the analyses of COBRA simulation results (84, 85) but have yet to be systematically integrated with the COBRA modeling of the human gut microbiota.

### Data integration.

High-throughput omics data can be used to contextualize genome-scale metabolic models (86, 87). Similarly, omics data from microbiome studies can be integrated into COBRA community microbiota models to generate context-specific models. For instance, in our recently published resource of gut microbe reconstructions (25), we have mapped metagenomic data of microbial abundances onto our set of microbes, which resulted in microbiota models consisting of about 100 microbes, indicating the need of comprehensive and scalable modeling approaches to analyze this data. Additionally, relative microbial abundances estimated from metagenomic data can be used to calibrate the microbial community (50). As such, the microbiota models are sample and/or person specific and permit the assessment of microbial capabilities unique to an individual, or a patient group. However, attention should be paid to the biological interpretation of this integration in different community modeling approaches. In compartment-based models, the bacterial abundances can be scaled with the community biomass (50, 81), which assumes that the microbial abundance correlates with the weight (dry weight) of each organism. In contrast, in individual-based models, the cell count of the species populations is scaled by the microbial abundances (77). As an alternative measure, the replication rates can be estimated from metagenomic data (88) and used as a proxy for microbial growth rates. If available, further data, such as meta-transcriptomic, meta-proteomic, and meta-metabolomic data (89), could be integrated into microbial community models to further constrain the models, thereby making them more representative of the real biological system.

### Model validation.

Experimental validation of community simulation results plays an important role to assess the predictive potential of the microbial community models. While community models can give interesting novel hypotheses with their simulation, particular attention should be paid to the biological relevance of the predictions. It is therefore important to relate and validate at least part of the simulations with experimental values that come from existing knowledge or direct experiments. Existing knowledge can be used to validate the predictions of community models, e.g., by comparing simulated with measured metabolite concentrations (63). This will also help to assess the relevance of the community models and how they should be interpreted. Since models of the human gut microbiota can be quite complicated and extensive, it is also difficult to find appropriate data or design experiments that can be used for validation. Part of the simulation results will thus be a novel hypothesis that can be validated only with new experiments. This can guide the targeted design of experimental studies, which becomes important in the field of the human gut microbiota to reduce the complexity to simple findings.

## CONCLUSIONS AND FUTURE PERSPECTIVES

The introduced COBRA community modeling approaches are promising tools to give novel insights and hypotheses of microbial consortia in the human gut. The models allow for the integration of multidimensional omics data and go beyond the descriptive nature of more traditional high-throughput data analysis approaches with the potential to unravel novel mechanisms. The different discussed modeling paradigms rely on various assumptions that need to be assessed before starting an analysis. On the basis of this assessment, the model that is least complicated and most explanatory for a specific research question should be chosen. It could also be fruitful to combine different approaches to see their consistency or potential differences for a specific problem. For example, it would be interesting to model single isolated species and communities separately to identify beneficial or detrimental effects on the single species level. Further comparisons can be made between network-based and modeling approaches. Can, for example, the negative and positive interactions in a coexistence correlation network be explained by the underlying metabolic interactions? Comparisons between algorithms could give further hints on candidate experiments that can be performed for validation. This can drive further research on the human microbiota, mechanistically predicting different treatments for various diseases.
